# Formation of nanoparticles by cooperative inclusion between (*S*)-camptothecin-modified dextrans and β-cyclodextrin polymers

**DOI:** 10.3762/bjoc.11.14

**Published:** 2015-01-21

**Authors:** Thorbjørn Terndrup Nielsen, Catherine Amiel, Laurent Duroux, Kim Lambertsen Larsen, Lars Wagner Städe, Reinhard Wimmer, Véronique Wintgens

**Affiliations:** 1 Department of Biotechnology, Chemistry and Environmental Engineering, Aalborg University, Sohngaardsholmsvej 57, 9000 Aalborg, Denmark; 2 Systèmes Polymères Complexes, ICMPE, CNRS and University Paris Est, 2 rue Henri Dunant, 94320 Thiais, France

**Keywords:** (*S*)-camptothecin, cyclodextrins, fluorescence, nanoparticles, ITC

## Abstract

Novel (*S*)-camptothecin–dextran polymers were obtained by “click” grafting of azide-modified (*S*)-camptothecin and alkyne-modified dextrans. Two series based on 10 kDa and 70 kDa dextrans were prepared with a degree of substitution of (*S*)-camptothecin between 3.1 and 10.2%. The binding properties with β-cyclodextrin and β-cyclodextrin polymers were measured by isothermal titration calorimetry and fluorescence spectroscopy, showing no binding with β-cyclodextrin but high binding with β-cyclodextrin polymers. In aqueous solution nanoparticles were formed from association between the (*S*)-camptothecin–dextran polymers and the β-cyclodextrin polymers.

## Introduction

Cancer remains to be the major cause of mortality in the western world and approximately one out of four deaths is currently due to cancer [1]. Conventional chemotherapy is associated with severe adverse effects by the non-specific action of anticancer agents. Furthermore, many of highly promising novel anticancer agents fail to even reach clinical trials simply due to limited aqueous solubility and thereby low bioavailability [2]. Hence, there is an urgent need for novel methods to overcome the solubility issue of these drugs and to ensure efficient transport to the desired site of action with limited adverse effects. In this respect nanoparticles have shown tremendous potential as drug-delivery vehicles due to their ability to encapsulate drugs and ensure delivery to specific tissues and organs. Nanoparticles in the size range of 50–200 nm have shown very promising results due to their ability to penetrate and accumulate in the highly porous cancer tissue with minimal impact on healthy tissue [2]. Furthermore, because of low renal clearance, the circulation time in the body is dramatically increased compared to conventional chemotherapeutic drugs whereby a drastic decrease in the dose needed for effective therapy can be realized. This specific delivery of drugs to the desired place of action lowers the adverse effects associated with conventional chemotherapy tremendously [3]. Therefore, much effort is continuously being made to develop novel drug-carrier systems. (*S*)-Camptothecin (CPT) is a natural cytotoxic quinoline alkaloid with a potent anticancer activity by inhibition of topoisomerase II. Its major drawback is its extremely low aqueous solubility of 2.5 µg/mL leading to a low bioavailability and it is thus a considerable formulation challenge [4]. Furthermore, at physiological conditions its active lactone form is hydrolysed to the inactive and toxic carboxylate form.

A way to circumvent these drawbacks can be to conjugate the CPT to hydrophilic polymers and hereby creating a water-soluble polymeric CPT prodrug whereby the apparent aqueous solubility of the drug is increased. Furthermore, modification of the CPT hydroxy group can lead to enhanced stability [5]. An example of such an approach is Cerulean CRLX101, a polymeric CPT conjugate which is currently in clinical trials [6]. Albeit, the CPT polymers reported are potential drug-delivery systems for CPT, they do not by themselves form nanoparticles of the desired size. Interaction with suitable correspondent polymers with pendent cyclodextrins may form nanoparticles with the target properties. In recent years cyclodextrin (CD) polymer-based drug-delivery systems have attracted much attention in this field [7–8]. CDs are cyclic sugar molecules constituted of glucose units with a hydrophilic exterior and a hydrophobic interior. The hydrophobic interior makes them excellent for forming so-called inclusion complexes with a wide range of compounds which has made CDs useful in numerous applications in research and industry [9]. Inclusion complexes are defined as the entity formed when hydrophobic molecules are encapsulated (in a dynamic equilibrium) by the CD molecule leading to drastically altered properties. In the pharmaceutical industry CDs are used to increase aqueous solubility, dissolution rate and chemical stability of drugs, as well as to enhance drug permeability through biological membranes [9]. For instance, CDs and CD derivatives have been applied in both stabilization and solubility enhancement of CPT [10–12].

CD polymers are macromolecular structures bearing many CD moieties. This gives a number of advantages compared to native CDs. First of all, affinity for drugs is often greatly improved due to a cooperative effect arising from the proximity of the CDs linked to the same macromolecule [13]. More importantly, the macromolecular nature of the polymers gives the possibility to form complex multicomponent drug-delivery systems such as nanoparticles and micelles. Recently, the first delivery of siRNA in humans using CD polymers was reported [14] and currently two drug-delivery systems based on CD polymers have entered clinical phase II trials in cancer treatment [6]. The advantages of CD polymers can be exploited in preparation of nanoparticles. It has been shown that CD polymers can form nanoparticles when associated with various amphiphilic polymers such as dextran modified with fatty acid chains [15] or adamantane [16] in aqueous solution. Simply by mixing solutions of β-CD polymers and the hydrophobic-modified dextrans with a degree of substitution between 4.7 and 9.1% nanoparticles with sizes between 100–400 nm are formed instantly. The nanoparticle formation arises from inclusion formation between hydrophobic chains on the dextran and the CD moieties of the CD polymers. The vacant CD cavities in the nanoparticles can be loaded with hydrophobic molecules and are stable for several days [15].

Here we report an alternative approach where instead of using biological inactive side chains, e.g., adamantane, CPT is applied. This was achieved by the synthesis of two series of novel CPT-polymers by conjugation of CPT to dextran backbones. The CPT was bound to the dextran by a cleavable ester linkage that should be biodegradable in vivo. It is demonstrated how the binding with β-CD polymers is affected by the number of CPT units on the polymer backbone and how this association can lead to novel nanoparticles in the 100 nm range of potential biomedical interest.

## Results and Discussion

### CPT-dextrans

#### Synthesis

**Scheme 1:**
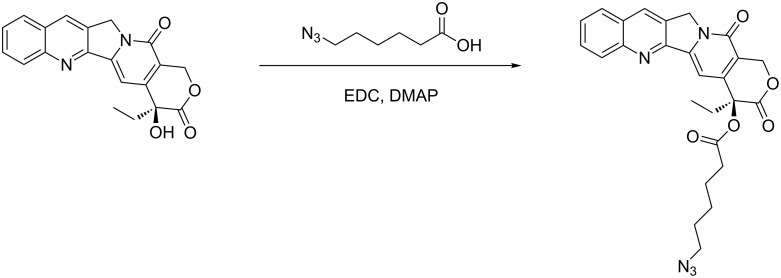
Reaction scheme for synthesis of azide-modified (*S*)-camptothecin.

The synthesis of CPT-dextrans was achieved in two steps. First azide-modified CPT (N_3_CPT) was synthesized by esterification with 6-azidohexanoic acid mediated by EDC/DMAP (Scheme 1). TLC showed complete conversion of the CPT after 24 hours. The N_3_CPT was purified by automated flash chromatography and obtained in good yields (>80%) and purity (verified by NMR spectroscopy).

**Scheme 2:**
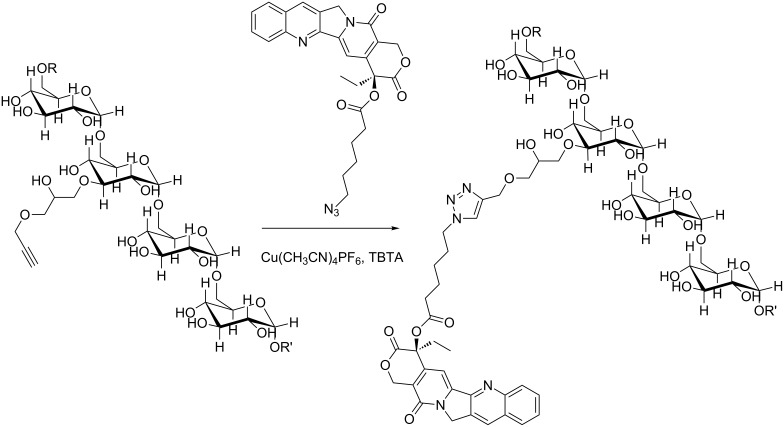
Cu(I)-catalyzed grafting of azide-modified (*S*)-camptothecin onto alkyne-modified dextran.

From the N_3_CPT two series of novel CPT-dextrans were synthesized by a copper(I)-catalyzed alkyne azide coupling (CuAAC). The dextrans were based on a 10 kDa and 70 kDa dextran backbone, respectively, which had been modified with alkynes by reaction with glycidyl propargyl ether (GP) as previously described [15]. The subsequent CuAACs with the alkyne-modified dextrans were made in DMSO using standard “click” conditions with 5 mol % Cu(I) compared to alkynes, stabilized with TBTA (Scheme 2). In order to maintain a reductive environment 15 mol % sodium ascorbate was added.

The “click” conjugation was achieved within 24 hours at ambient temperature. The complete conversion of terminal alkyne protons with corresponding appearance of the characteristic triazole signal at 7.99 ppm (H) and 126 ppm (C) was found by NMR spectroscopy. The degree of substitution (DS) was calculated from integration of the triazole signal compared to the integral of the anomeric signals of the glucose units of dextran at 4.68 ppm. Four CPT-dextrans from each backbone (10 kDa and 70 kDa) were obtained in good yields (85–94%) after purification by precipitation and trituration with acetone and dichloromethane. The DS was in the range of 3.1–10.2 mol %. For both the 10 kDa and 70 kDa series with a CPT content of up to approximately 9% (w/w) the aqueous solubility was fairly high (above 10 g/L). This is approximate a 360 times increase compared to unmodified CPT with a solubility of 2.5 µg/mL. Higher CPT contents lead to a dramatic decrease in solubility and dextrans with a CPT content above 15% (w/w) were almost completely insoluble in water (Table 1).

**Table 1:** DS, CPT content, number of CPT units per chain and aqueous solubility of the synthesized (*S*)-camptothecin-dextrans.

Compound	DS [mol %]^a^	% CPT [w/w]	CPT/chain	Aqueous solubility

D10GP-CPT1	4.6	8.7	2.8	++
D10GP-CPT2	6.1	11.1	3.8	++
D10GP-CPT3	7.3	12.9	4.5	+
D10GP-CPT4	9.2	15.6	5.7	−
D70GP-CPT1	3.1	6.0	13.4	++
D70GP-CPT2	4.5	8.6	19.9	++
D70GP-CPT3	6.1	11.0	26.4	+
D70GP-CPT4	10.2	16.7	43.2	−

^a^mol % compared to glucose units of the dextran backbone.

#### Binding properties and formation of nanoparticles

The ability to form inclusion complexes with β-CD and β-CD-polymers was determined by isothermal titration calorimetry (ITC) and fluorescence spectroscopy. The β-CD polymer (D70HPβ-CD) had been prepared from a 70 kDa dextran backbone modified with 6-heptynoic acid in a similar way as already described in literature [17] and had a β-CD content of 58.6% (w/w) (Supporting Information File 1). D10GP-CPT1 (DS 4.6) and D70GP-CPT2 (DS 4.5) were chosen since the similar DS should make comparison possible.

#### Isothermal titration calorimetry

The different thermodynamic parameters derived assuming a 1:1 complex formation between β-CD and CPT units are given in Table 2. The interactions between the polymers were in both cases exothermic. The apparent association constant (*K*_a_) is more than one order of magnitude higher for D70GP-CPT2 than D10GP-CPT1. The complex formation is enthalpy driven in the case of D10GP-CPT1, but mainly entropy driven (|*T*Δ*S*| > |Δ*H*|) in the case of D70GP-CPT2. This high positive entropy variation shows a cooperative inclusion of the polymers.

**Table 2:** ITC data for titration of D70HPβ-CD with D10GP-CPT1 and D70GP-CPT2, respectively.

Compound	*n*	*K*_a_ [M^−1^]	Δ*G*	Δ*H* [kJ/mol]	*T*Δ*S* [kJ/mol]

D10GP-CPT1	0.73	1030	−17.2	−22.3	−5.1
D70GP-CPT2	1.45	16700	−24.1	−8.6	15.5

No binding between native β-CD and D70GP-CPT2 could be measured by ITC.

**Figure 1:**
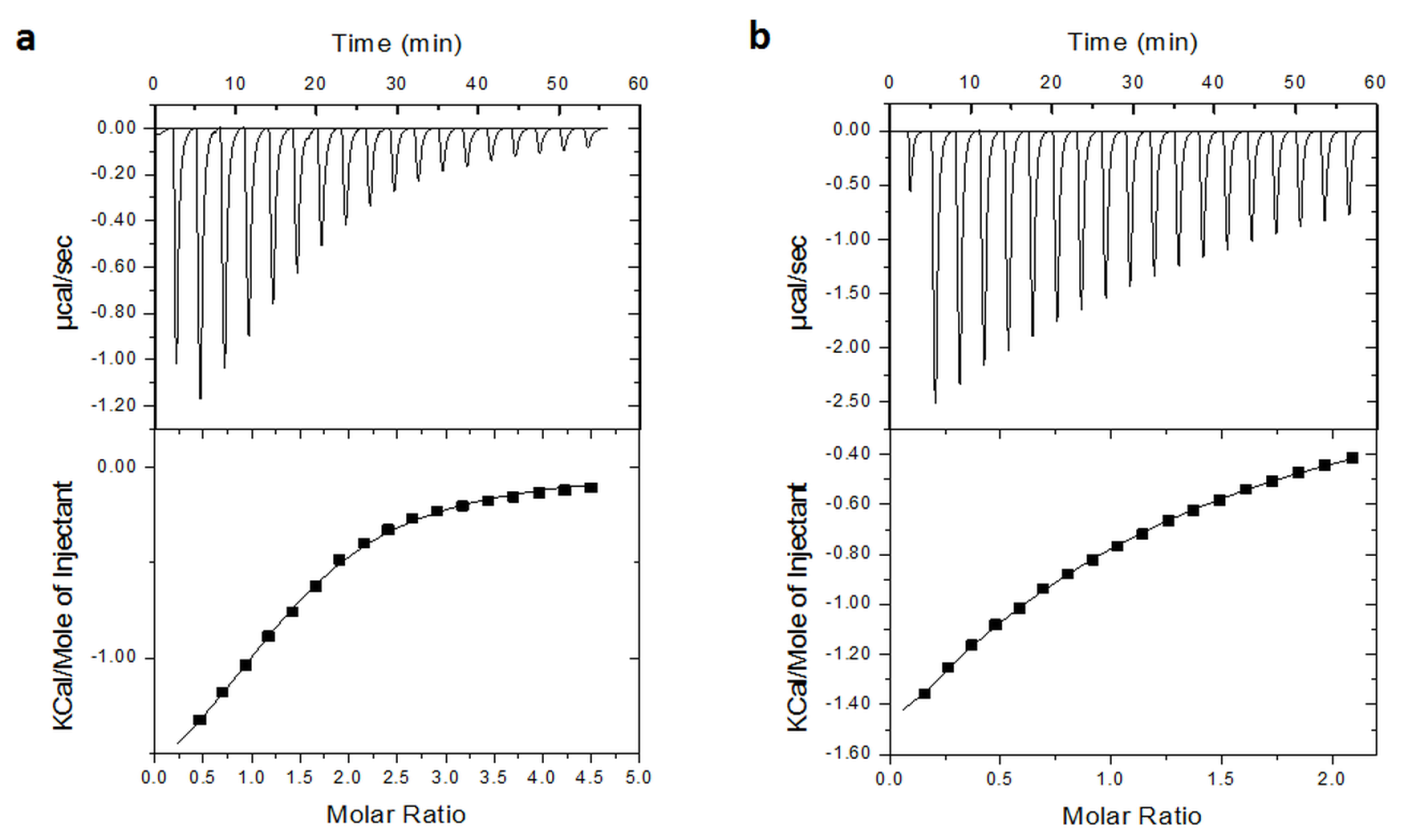
Titration of a) D70GP-CPT2 and b) D10GP-CPT1 by D70HPβ-CD at 298 K showing heat flow as a function of time and integrated heat vs the molar ratio (enthalpogram).

Figure 1 shows the heat flow curves and the enthalpograms obtained for the titration of D70GP-CPT2 and D10GP-CPT1 by D70HPβ-CD at 298 K.

#### Fluorescence spectroscopy

**Figure 2:**
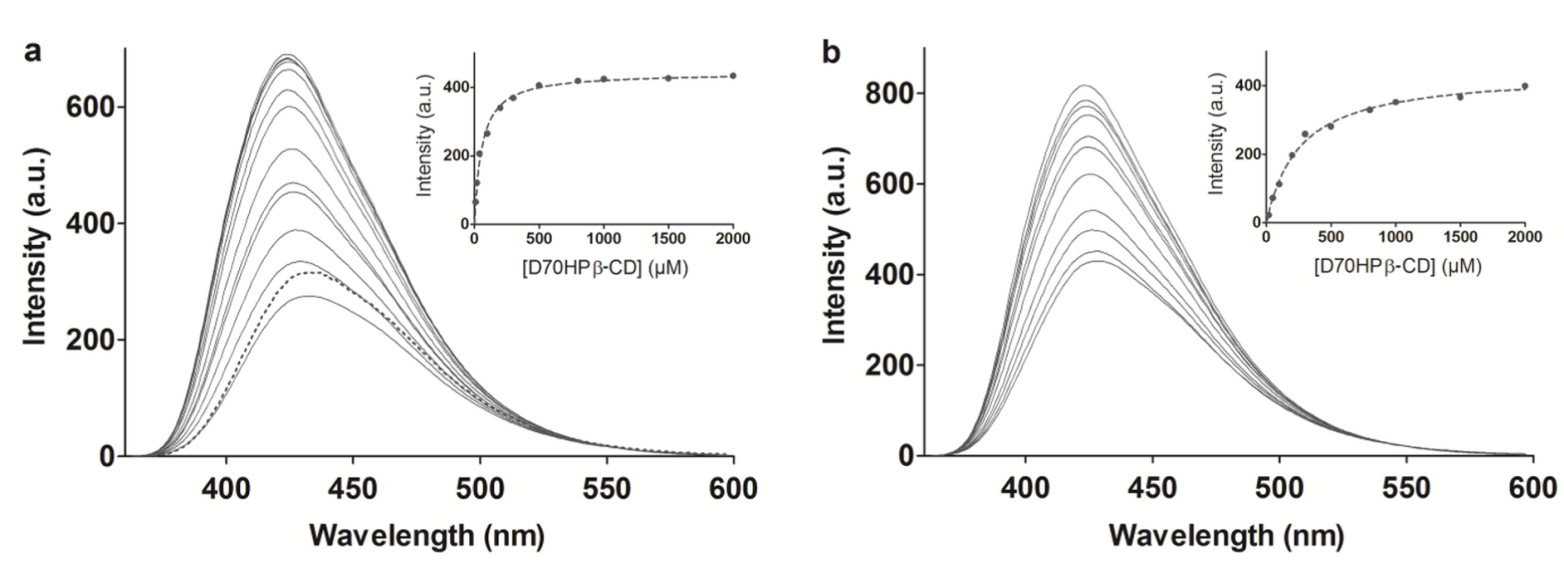
Fluorescence emission spectra of a) D70GPCPT and b) D10GPCPT recorded at different concentrations (0–2 mM β-CD) of D70HPβ-CD. The dotted line in a) is the emission spectra of the D70GPCPT polymer with 2 mM of free β-CD. The inserts are the variation of FI at 430 nm plotted as a function of D70HPβ-CD concentration, with FI of the CPT polymer solutions in the absence of D70HPβ-CD subtracted.

Figure 2 shows the fluorescence spectra of D70GP-CPT2 (a) and D10GP-CPT1 (b) recorded at different concentrations of D70HPβ-CD. For the CPT and 2 mM β-CD there is only a minor fluorescence intensity (FI) enhancement which is in accordance with the low association constant previously reported in literature in the range of 202–206 M^−1^ [10,12]. For both of the CPT polymers a vast increase in FI accompanied by an 8 nm blue shift of the emission wavelength in response to increased D70HPβ-CD concentration (0–2 mM) is observed. This is typical for a decrease in local dielectric constant (i.e., shielding from water solvent) and thereby an indicator of inclusion of the pendant CPT in the apolar β-CD cavities. *K*_a_ values were determined by plotting the variation in FI at 430 nm as a function of D70HPβ-CD concentration. The data was fitted to a binding isotherm yielding apparent *K*_a_ values of 18000 M^−1^ and 3900 M^−1^ for D70GP-CPT2 and D10GP-CPT1, respectively. These significant changes in association constants and the observation that addition of 2 mM β-CD to the polymers only yields a minor FI enhancement (dotted line Figure 2a) indicate a strong cooperative inclusion effect as a result of the increased polymer length, which is well in accordance with the ITC measurements.

Hence, the apparent *K*_a_ largely depends on the *M*_w_ of the dextran backbone to which the CPT is conjugated. Whereas a 10 kDa CPT dextran with a DS of 4.6 mol % contains 2.6 CPT moieties on average, the 70 kDa counterpart contains almost 20, leading to a significant increase in the number of anchoring points on each polymer chain. The difference of binding strength between a 10 kDa and a 70 kDa CPT-dextran illustrates the influence of the cooperative inclusion as described by Breslow et. al in 1995 [18].

#### Formation of nanoparticles

The influence of the backbone was not only reflected in the higher binding constant but also in the ability of the CPT-dextrans to form nanoparticles when associated with β-CD polymers. As illustrated in Table 3 the D70GP-CPT2 with a DS of 4.6 mol % forms highly monodisperse nanoparticles when associated with D70HPβ-CD in aqueous media, measured by dynamic light scattering (DLS). The optimum ratio between CPT and β-CD units was found to be 1:1 but even more extreme ratios of 1:4 and 4:1 led to the formation of nanoparticles. For the 1:1 complexes the concentrations of CPT and β-CD units were 2.25·10^−4^ M after mixture corresponding to a CPT content of 78 µg/mL. The nanoparticles were stable for more than three days. No monodisperse nanoparticles were formed when associated with D70HPβ-CD, probably due to the much lower solubility of D70GP-CPT3 (DS 6.1%). However, D70GP-CPT1 (DS 3.1%) led to formation of nanoparticles but of larger size (≥200 nm) and lower stability. None of the D10GP-CPTs was able to form nanoparticles. The formation of nanoparticles is expected to significantly increase the stability of the CTP lactone, due to the protection from the aqueous environment.

**Table 3:** Development in size over time of nanoparticles formed by association between D70GP-CPT2 and D70HPβ-CD at different ratios, measured by dynamic light scattering.

CPT:β-CD	Hours	Z (nm)	PDI

1:1	0.5	114.7	0.069
1:1	2	116.4	0.063
1:1	8	117.5	0.073
1:1	24	111.8	0.102
1:1	48	112.3	0.084
1:1	72	99.9	0.163
1:4	0.5	93.4	0.101
1:4	2	94.4	0.076
1:4	8	96.6	0.096
1:4	24	75.8	0.182
1:4	48	75.9	0.152
1:4	72	55.2	0.273
4:1	0.5	84.4	0.143
4:1	2	86.2	0.151
4:1	8	86.9	0.110
4:1	24	81.2	0.145
4:1	48	64.7	0.254
4:1	72	59.0	0.290

## Conclusion

Novel (*S*)-camptothecin-dextran polymers were obtained by “click” grafting of azide-modified (*S*)-camptothecin and alkyne-modified dextrans. Two series based on 10 kDa and 70 kDa dextrans were prepared with a DS of (*S*)-camptothecin between 3.1 and 10.2%. It was measured by ITC and fluorescence spectroscopy that the binding properties with β-CD polymers depended significantly on the molecular weight of the dextran backbone of the (*S*)-camptothecin-dextran polymers, where the strongest binding was observed with the 70 kDa (*S*)-camptothecin-dextran polymers due to a cooperative effect. No binding with native β-CD could be measured. In aqueous solution two of the 70 kDa (*S*)-camptothecin-dextran polymers formed nanoparticles when associated with β-CD-polymers.

## Experimental

6-Azidohexanoic acid [19] and tris[(1-benzyl-1*H*-1,2,3-triazol-4-yl)methyl]amine (TBTA) [20] were prepared according to literature. (*S*)-Camptothecin >95.0% was obtained from TCI Japan. Ambersep GT74 resin, anhydrous dichloromethane (DCM) ≥99.8%, *N*-(3-dimethylaminopropyl)-*N*′-ethylcarbodiimide hydrochloride (EDC) ≥99.0%, 4-(dimethylamino)pyridine (DMAP) ≥98%, anhydrous dimethyl sulfoxide (DMSO) ≥99.5%, sodium L-ascorbate (NaAsc) ≥98%, tetrakis(acetonitrile)copper(I) hexafluorophosphate (Cu(CH_3_CN)_4_PF_6_) 97% were all from Sigma-Aldrich and were used as received. A β-CD-dextran polymer (D70HPβ-CD) was obtained by grafting 6-azido-*O*-deoxyβ-CD onto 70 kDa dextran modified by 7-heptynoic acid, in a similar way as described in literature [15]. The polymer contained 58.6% β-CD (w/w). TLC analyses were made with ALUGRAM^®^ SIL G/UV_254_ TLC plates with 0.2 mm silica gel, and visualized using UV light. Automated flash chromatography was made on a Grace Davidson Discovery Science Revelaris system. NMR spectra were recorded on a Bruker AVIII-600 spectrometer with a 5 mm TCI (H–C/N–D) probe at 298 K for samples in CDCl_3_ and 310 K for samples in DMSO-*d*_6_. ITC measurements were carried out at 25 °C using a MicroCal VPITC microcalorimeter. In each titration, injections of 5 μL of D70HPβ-CD solution (2.5·10^−3^ M) were added from the computer-controlled 295 μL microsyringe at an interval of 180 s into the cell (volume 1.4569 mL) containing the polymer solution (corresponding to 1·10^−4^ M of CPT), while stirring at 450 rpm. The raw experimental data were obtained as the amount of heat produced per second following each injection of CPT as a function of time. Integration of the heat flow peaks by the instrument software (after taking the heat of dilution into account) gives the amount of heat produced per injection. The experimental data were fitted with a theoretical titration curve using the instrument software, assuming a 1:1 complex. The enthalpy change, Δ*H*, the binding constant (*K*), and the stoichiometry (*n*), were the adjustable parameters. Fluorescence emission spectra were recorded on Varian Cary Eclipse fluorescence spectrophotometer. For each sample an average of 5 scans was recorded, with excitation and emission slits set to 5 nm and a PMT detector voltage of 400 V. The excitation wavelength was set at 350 nm and wave scans recorded between 360 nm and 600 nm. For the estimation of association constants by steady-state fluorescence spectroscopy, stock solutions of D70GPCPT and D10GPCPT (45 µM CPT) and D70HPβ-CD (2 mM) were prepared in MQ water. Titration was performed by diluting the D70HPβ-CD stock solution to its final concentration (0–2 mM) with the corresponding CPT polymer, while ensuring a constant concentration of CPT polymer (45 µM). Nanoparticles were prepared by mixing 0.6 mL of the CPT-dextran solutions with 0.6 mL D70HPβ-CD solutions at room temperature under magnetic stirring at 150 rpm. The mean hydrodynamic diameter and the polydispersity index (PDI) of the nanoparticles were determined by dynamic light scattering (DLS) using a Malvern Instruments Zetasizer Nano ZS equipped with a He–Ne laser (633 nm, scattering angle 173°). Each sample was measured ten times for ten seconds at 25 °C.

### Syntheses

#### N_3_CPT

CPT (500 mg, 1.434 mmol) and 6-azidohexanoic acid were dissolved in 75 mL DCM. DMAP (89 mg, 0.715 mmol) and EDC (982 mg, 5.02 mmol) were added and the solution was stirred at rt overnight. TLC showed complete conversion of the CPT and the solution was washed with water (3 × 100 mL), brine (100 mL) and dried over magnesium sulfate. The solution was filtered, concentrated to approx. 10 mL in vacuo and subjected to automated flash chromatography on a 40 g silica column using a linear DCM/methanol gradient (0–8%). The appropriate fractions were combined, the solvent was removed in vacuo and 575 mg (82%) yellow powder was isolated. ^1^H NMR (CDCl_3_) δ (ppm) 8.41 (s, 1H), 8.22 (d, 1H), 7.95 (d, 1H), 7.85 (t, 1H), 7.68 (t, 1H), 7.21 (s, 1H), 5.68 (d, 1H), 5.42 (d, 1H), (dd, 2H), 3.22 (m, 2H) 2.52 (m, 2H), 2.28 (m, 1H), 2.16 (m, 1H), 1.69 (m, 2H), 1.60 (m, 6H), 1.43 (m, 2H), 0.98 (t, 3H).

#### CPT-dextrans

D10GP or D70GP (200 mg, 0.037–0.114 mmol alkyne) and N_3_CPT (1.25 equiv) were dissolved in 30 mL degassed DMSO. TBTA (0.055 equiv) and NaAsc (0.15 equiv) were added and the solution was degassed with nitrogen for 5 minutes. Cu(CH_3_CN)_4_PF_6_ (0.05 equiv) was added and nitrogen was bubbled through the solution for additional 5 minutes. After stirring the solution under nitrogen for 24 hours at rt the CPT-dextrans were precipitated in 300 mL acetone and filtered. The products were successively washed with DCM and acetone and dried in vacuo. 207–251 mg (85–94%) yellow powder was isolated. ^1^H NMR (DMSO-*d*_6_) δ (ppm) 8.69 (s, 1H), 8.13 (s, 1H), 8.12 (s, 1H), 7.99 (s, 1H), 7.84 (s, 1H), 7.71 (s, 1H), 7.06 (s, 1H), 5.48 (s, 2H), 4.86 (s, 1H), 4.78 (s, 1H), 4.68 (s, 1H), 4.48 (s, 2H), 4.42 (s, 1H), 4.28 (s, 2H), 3.99-2.96 (m, 9H), 2.52 ( s, 2H), 2.15 (s, 2H), 1.83 (s, 2H), 1.60, (s, 2H), 1.31 (s, 2H), 1.23 (s, 1H), 0.91 (s, 3H).

## Supporting Information

File 1 ^1^H NMR and HSQC spectra of N_3_CPT and D70GP-CPT2 and ITC data from titration of D70GP-CPT with native β-CD.
