# Supplementary material for: Formation of nanoparticles by cooperative inclusion between (S)-camptothecin-modified dextrans and β-cyclodextrin polymers
Source: Beilstein J Org Chem. 2015 Jan 21;11:147–54. doi: 10.3762/bjoc.11.14 (PMC4311650; doi:10.3762/bjoc.11.14)
Supplement: File 1 — 1H NMR and HSQC spectra of N3CPT and D70GP-CPT2 and ITC data from titration of D70GP-CPT with native β-CD. [file Beilstein_J_Org_Chem-11-147-s001.pdf]

Supporting Information
for
Formation of nanoparticles by cooperative inclusion between (*S*)-camptothecin-modified dextrans and β-cyclodextrin polymers

Thorbjørn Terndrup Nielsen*[1], Catherine Amiel[2], Laurent Duroux[1], Kim Lambertsen Larsen[1], Lars Wagner Städe[1], Reinhard Wimmer[1] and Véronique Wintgens[2]

Address: [1]Department of Biotechnology, Chemistry and Environmental Engineering, Aalborg University, Sohngaardsholmsvej 57, 9000 Aalborg, Denmark and [2]Systèmes Polymères Complexes, ICMPE, CNRS and University Paris Est, 2 rue Henri Dunant, 94320 Thiais, France

Email: Thorbjørn Terndrup Nielsen - ttn@bio.aau.dk
*Corresponding author

^{1}H NMR and HSQC spectra of N_3CPT and D70GP-CPT2 and ITC data from titration of D70GP-CPT with native β-CD

Experimental

6-Heptynoyl chloride

6-heptynoic acid (0.83 g, 6 mmol) was dissolved in 5 mL dry DCM and added a catalytic amount of DMF (1μL). The solution was cooled in an ice bath and oxalyl chloride (0.572 mL, 6.6 mmol) was added atmosphere. The solution was stirred at ambient temperature for 3 hours. DCM was removed *in vacou and* the heptynoyl chloride was used immediately in the next step without purification.

D70HP

Lithium chloride (0.75 g) and dextran (3 g, 18.5 mmol glucose units) were dissolved in 75 mL DMF by warming. DMAP (2.2 g, 1.8 mmol) was added. Freshly prepared 6-heptynoyl chloride (0.87 g, 6 mmol) was dissolved in 5 mL of DMF and added the solution of dextran at ambient temperature. The temperature was raised to 80° C and the solution was stirred for two hours under nitrogen. After cooling to ambient temperature the product was precipitated in 500 mL 2-PrOH, filtered and washed with 200 mL 2-PrOH. After drying under nitrogen, the product was dissolved in 100 mL water and dialyzed against water for 36 hours. The product was obtained by freeze drying as white solid. The DS of heptynoic acid was determined by ^{1}H-NMR (22.7%).

D70HPβ-CD

0.75 g of D70HP (0.92 mmol alkyne), N_3βCD (1.86 g, 1.6 mmol) and tris-(benzyltriazolylmethyl)amine (28 mg, 0.05 mmol) were dissolved in 75 mL degassed DMSO/H2O 1:1 under nitrogen atmosphere. Sodium ascorbate (27 mg, 0.13 mmol) was added and the solution was degassed by ultrasound and subsequent bubbling with nitrogen for five minutes while reaching 50 °C. Copper(II)sulfate (7.3 mg, 0.045 mmol) was added and the solution was stirred for 24 hours and subsequently dialyzed against water for 72 hours. The solution was swirled over seven grams of Ambersept GT74 resin for 24 hours, filtered and freeze dried.

Estimation of association constants by steady-state fluorescence.

The apparent association constants were determined by plotting the variation in fluorescence intensity (FI) at 430 nm as a function of D70HXβCD concentration (0 – 2 mM). The data was fitted by non-linear regression in Graphpad *v. 5.0* to a binding isotherm (rectangular hyperbola) on the equation:

$$Y = \frac{B_{max} * X}{K_d * X}$$

Here B_{max} is the maximum specific binding expressed in the same units as the Y-axis. K_d is the disassociation constant (the inverse of K_a) expressed in the same units as the X-axis.

Association constants for the CPT polymers and free βCD were too low to be determined i.e. βCD concentration required for saturation of CPT moieties not within the βCD solubility range.

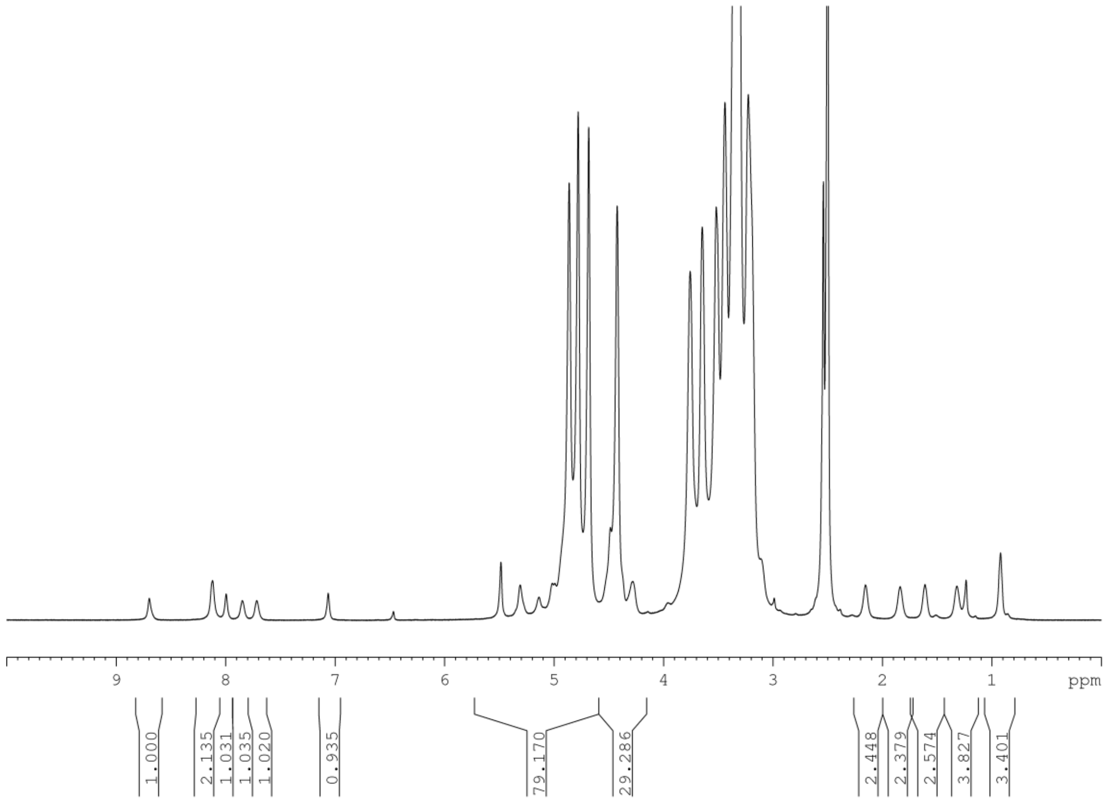

Figure S1: ^{1}H NMR spectrum of D70GP-CPT2 (DMSO-d_6)

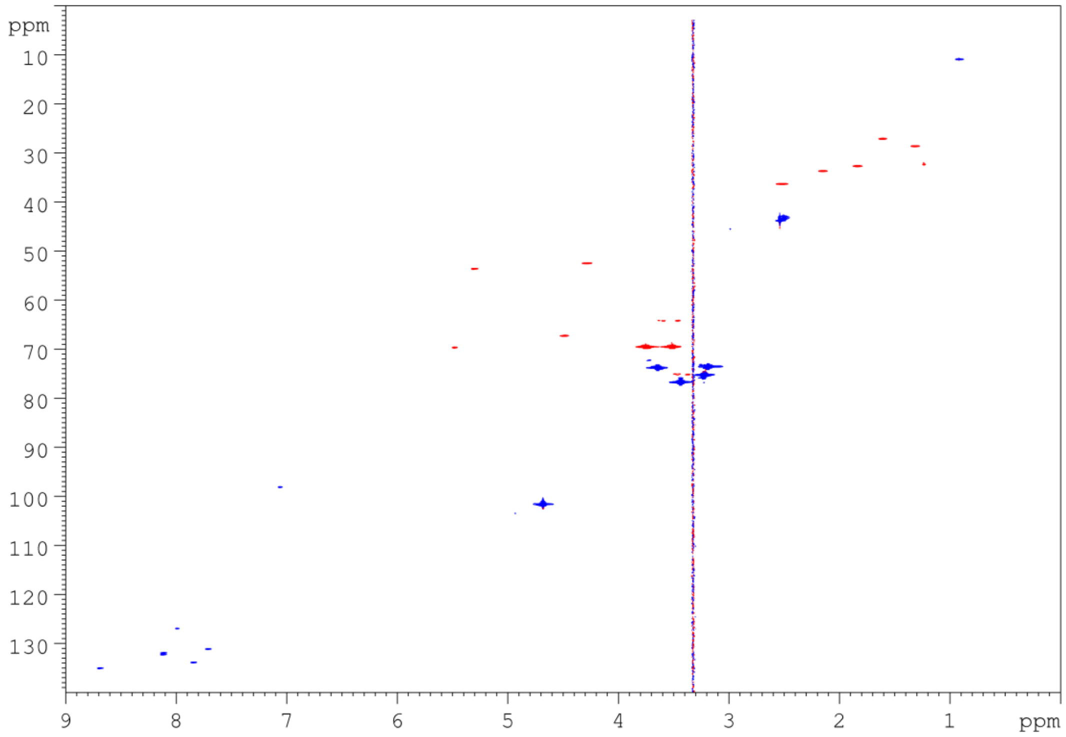

Figure S2: Multiplicity edited HSQC spectrum of D70GP-CPT2 (DMSO-d_6)

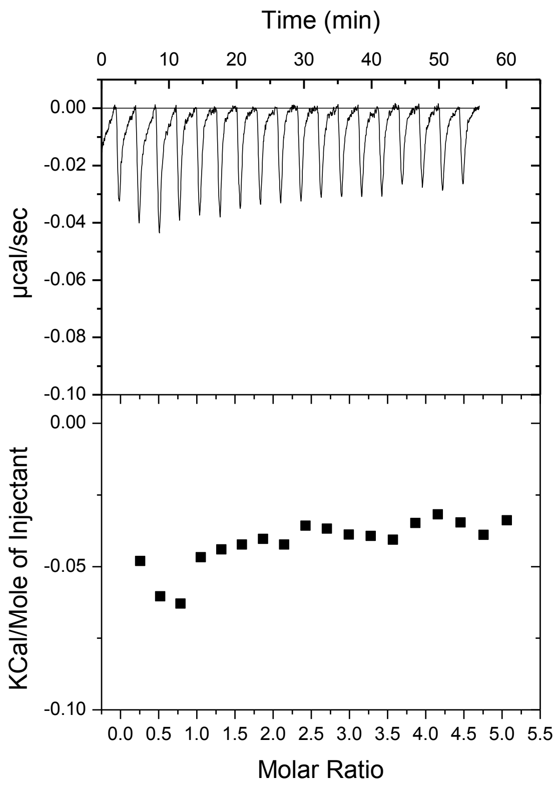

Figure S3: Heat flow curves and the enthalpograms obtained for the titration of D70GP-CPT2 by βCD.

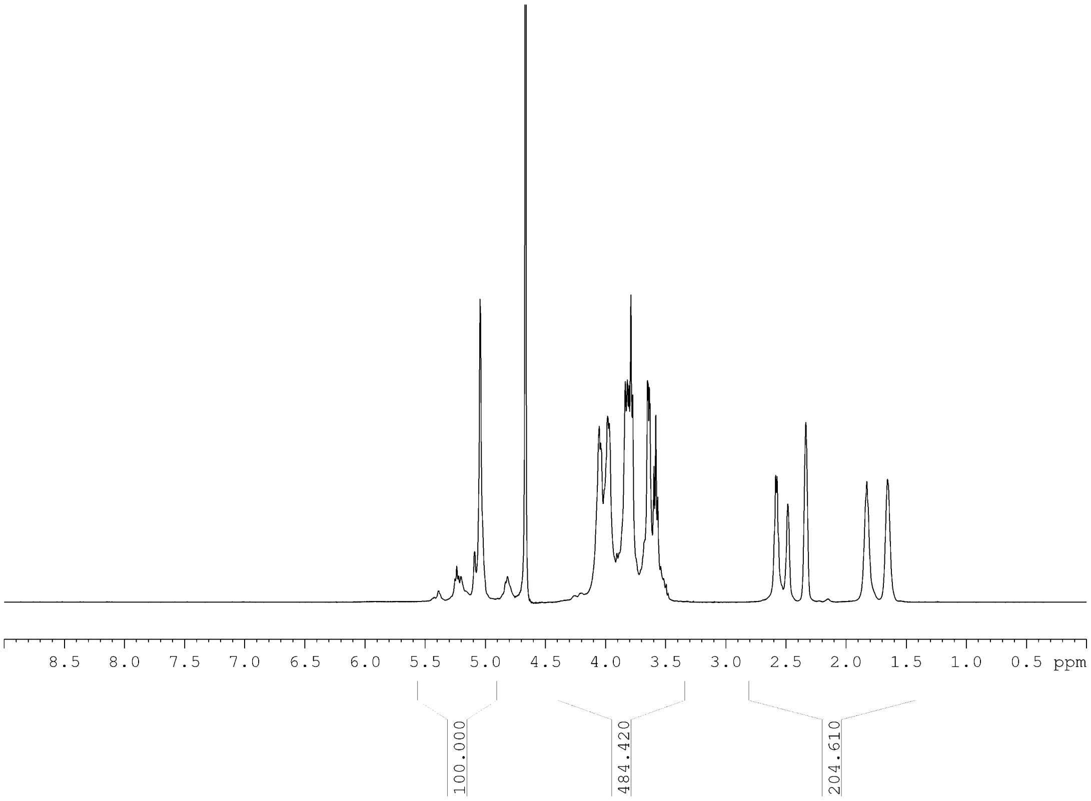

Figure S4: ^{1}H-NMR spectrum of D70HP (D_2O)

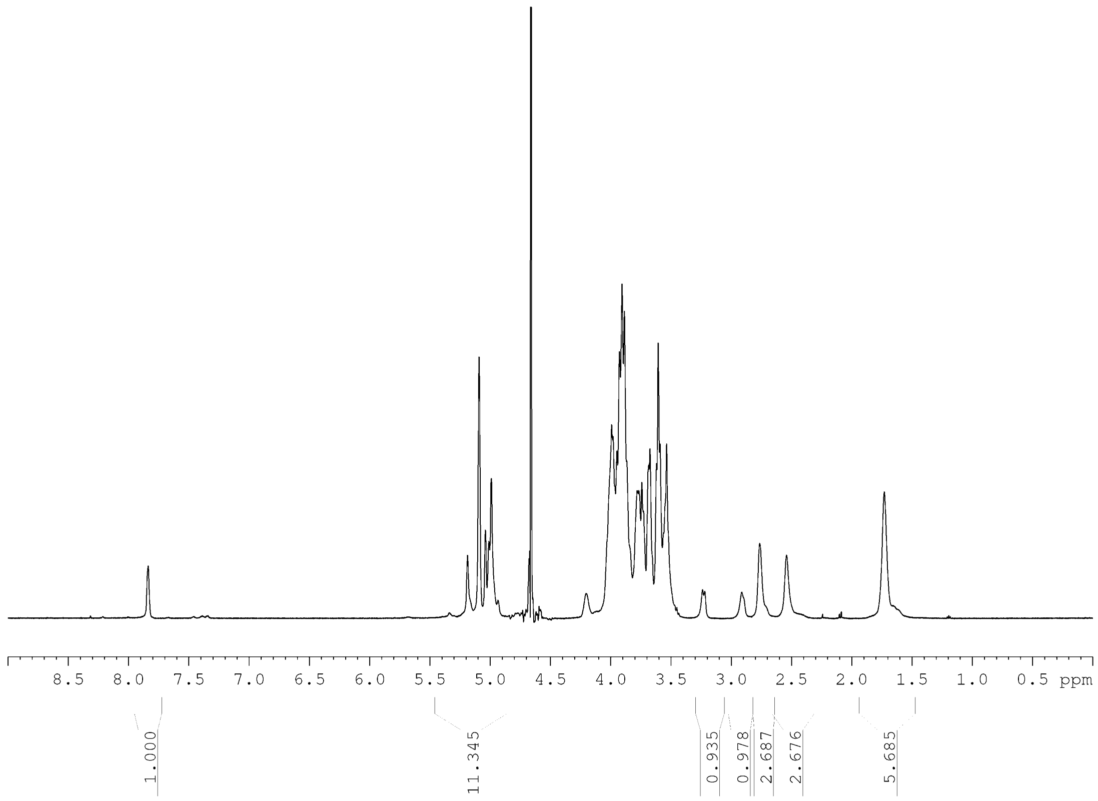

Figure S5: ^{1}H-NMR spectrum of D70HPβCD (D_2O)
